# Clinical Predictors of Time to Hospital Discharge in Children With New‐Onset Diabetic Ketoacidosis in Zambia: A Retrospective Cohort Study

**DOI:** 10.1002/edm2.70308

**Published:** 2026-08-12

**Authors:** Gunet Mwalungali, Blessings Kaimbo, Martin Kampamba, Larry Mweetwa, Christine Mutati, Chanda Chisunka, Annie Silwimba, Jimmy Hangoma

**Affiliations:** ^1^ School of Medicine Cavendish University Lusaka Zambia; ^2^ Department of Pharmacy University of Zambia Lusaka Zambia; ^3^ Ministry of Technology and Science Government of the Republic of Zambia Lusaka Zambia; ^4^ School of Applied and Health Sciences, Pharmacy Department Evelyn Hone College of Applied Arts and Commerce Lusaka Zambia; ^5^ Department of Pharmacy, School of Health Sciences Levy Mwanawasa Medical University Lusaka Zambia

**Keywords:** child, diabetic ketoacidosis, hospitalised, type 1 diabetes mellitus

## Abstract

**Background and Aims:**

Diabetic ketoacidosis (DKA) at type 1 diabetes diagnosis is common in low‐resource settings and associated with prolonged hospitalisation. We aimed to identify determinants of time to discharge among children with new‐onset DKA in Zambia.

**Methods:**

We conducted a retrospective cohort study of children aged 0–16 years with newly diagnosed T1DM and DKA at the University Teaching Hospital, Lusaka (2018–2023). The primary outcome was time to discharge (days). We used Kaplan–Meier with log‐rank tests and Cox regression. To address proportional hazards (PH) violation, we fitted a stratified Cox model (stratified by HbA1c, vomiting and DKA severity) for PH validation and a granulated Cox model with DKA severity (mild, moderate, severe) to estimate independent effects. PH was verified using Schoenfeld residuals.

**Results:**

Among 353 children (mean age 8.3 years, SD 4.6), median stay was 5 days (IQR 4–8). Log‐rank tests showed significant differences in discharge probability by DKA severity (χ^2^ = 70.32, *p* < 0.001) and vomiting (χ^2^ = 30.35, *p* < 0.001). In the granulated model, DKA severity was the strongest predictor of delayed discharge (moderate: HR = 0.01, *p* < 0.001; severe: HR = 0.01, *p* < 0.001), while infection was associated with faster discharge (HR = 1.51, *p* < 0.001). Ketonuria lost significance after DKA adjustment (HR = 0.90, *p* = 0.770), indicating a marker not an independent predictor. The stratified model confirmed PH satisfaction (global test χ^2^ = 5.62, df = 7, *p* = 0.584). Age, sex, polyuria, family history and residence were not significant.

**Conclusions:**

DKA severity is the strongest independent predictor of prolonged hospital stay in children with new‐onset DKA. Ketonuria is a useful bedside marker but lacks independent effect beyond severity. Infection independently predicts faster discharge. These findings could inform risk stratification and resource allocation in similar settings.

## Graphical abstract

In children with new‐onset diabetic ketoacidosis in Zambia, severity strongly predicts prolonged hospital stay, while infection paradoxically shortens discharge time. Ketonuria is a marker of severity but not an independent predictor.

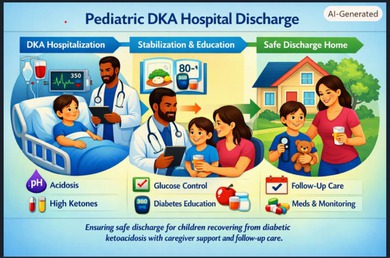

## 1 Introduction

The global incidence of childhood type 1 diabetes has increased by over 30% in the past three decades, with an estimated 108,300 children under 15 years diagnosed annually worldwide [1]. The burden of disease is disproportionately high in low‐ and middle‐income countries, where rates of diabetic ketoacidosis at diagnosis often exceed 60%–70% [2]. Diabetic ketoacidosis (DKA) characterised by hyperglycaemia, ketosis and metabolic acidosis remains the most serious acute presentation of T1DM and is the leading cause of hospitalisation and death in newly diagnosed children [3, 4].

In high‐income countries, DKA at T1DM diagnosis occurs in 15%–30% of children, but in low‐ and middle‐income countries (LMICs), rates often exceed 60%–70% [5, 6]. Zambia, like many African nations, faces challenges of delayed diagnosis, limited access to insulin and inadequate paediatric diabetes services, resulting in a high burden of moderate and severe DKA at presentation [7, 8].

Prolonged hospital stay in DKA increases the risk of iatrogenic complications, consumes scarce healthcare resources and places financial strain on families [9, 10]. Despite this, few studies from LMICs have systematically evaluated predictors of time to discharge using appropriate time‐to‐event methods with verification of model assumptions [11, 12].

We therefore conducted a retrospective cohort study using Kaplan–Meier methods with log‐rank testing and Cox proportional hazards regression with formal testing of the proportional hazards assumption to identify determinants of time to hospital discharge in children with new‐onset DKA at Zambia's largest tertiary referral hospital.

## 2 Materials and Methods

### 2.1 Study Design and Setting

We performed a retrospective cohort study at the University Teaching Hospital (UTH) in Lusaka, Zambia. UTH is the national tertiary referral centre for paediatric endocrinology, serving an estimated catchment of over 2 million children from both urban and rural districts. The study period was 1 January 2018 to 31 December 2023.

### 2.2 Participants

We included all children aged 0–16 years who:

- Were newly diagnosed with T1DM between 1 January 2018 and 31 December 2023:
- Had documented DKA at presentation (based on ISPAD criteria: hyperglycaemia > 11.1 mmol/L, venous pH < 7.3 or bicarbonate < 15 mmol/L and ketonaemia/ketonuria) [3];
- Had complete admission and discharge records

We excluded children with pre‐existing diabetes, incomplete records for key variables or transfer to another facility before discharge.

### 2.3 Outcome

The primary outcome was time to hospital discharge (days), defined as the interval from admission to discharge order. All patients were discharged (no in‐hospital deaths or transfers out), so no censoring occurred.

### 2.4 Variables

Data were extracted from medical records using a standardised form. Variables included:

- **Demographic**: age (years), sex (male/female), residence (urban/rural). Age groups were categorised as follows: ≤ 5 years, 6–10 years (including patients aged 5.1–5.9 years) and 11–16 years (including patients aged 10.1–10.9 years).
- **Clinical**: presence of vomiting (yes/no), polyuria (yes/no), infection at presentation (yes/no), family history of T1DM (yes/no). Infection at presentation was defined based on documented clinical criteria, including fever (temperature ≥ 38.0°C), clinical signs and symptoms consistent with infection (e.g., cough, tachypnoea, dysuria, localising signs) and supportive laboratory findings (elevated white blood cell count > 11.0 × 10^9^/L and/or C‐reactive protein > 5 mg/L) where available. Infection severity was not consistently graded in the medical records, limiting our ability to examine the relationship between infection severity and length of hospital stay
- **Laboratory**: HbA1c (%), ketonuria (positive/negative)
- **DKA severity**: categorised as mild (0), moderate (1) or severe (2) based on ISPAD guidelines [3]. All patients were managed according to a standardised DKA protocol adapted from ISPAD guidelines, which includes intravenous fluid resuscitation, continuous insulin infusion (0.1 units/kg/h) and electrolyte replacement. Patients with severe DKA (pH < 7.1) or altered consciousness were admitted to the paediatric ICU, while the majority were managed on the general paediatric ward. However, the duration of ICU versus ward stay was not consistently documented

### 2.5 Statistical Analysis

We summarised baseline characteristics using means (SD) or medians (IQR) for continuous variables and frequencies (%) for categorical variables.

#### 2.5.1 Kaplan–Meier Analysis With Log‐Rank Test

Kaplan–Meier survival curves were plotted for discharge probability by DKA severity (mild vs. moderate/severe) and by vomiting status. Differences between groups were formally tested using the log‐rank test, with *p* < 0.05 considered statistically significant.

#### 2.5.2 Cox Proportional Hazards Regression

We fitted a Cox proportional hazards regression model with the following prespecified covariates: sex, age, HbA1c, family history of T1DM, polyuria, vomiting and DKA severity (mild, moderate and severe), ketonuria, infection at presentation and residence.

#### 2.5.3 Testing the Proportional Hazards Assumption

The proportional hazards assumption was tested using Schoenfeld residuals in Stata 18. A non‐significant global test (*p* > 0.05) supports the PH assumption.

#### 2.5.4 Addressing Violation of the Proportional Hazards Assumption

Since the global Schoenfeld test was significant for the standard Cox model (*p* < 0.001), with individual violations for HbA1c (*p* = 0.0016), vomiting (*p* < 0.001) and DKA severity (*p* = 0.0012), we fitted two complementary models:

1. **Stratified Cox model**: Stratified by HbA1c categories (tertiles: ≤ 9.5%, 9.6%–13.0%, > 13.0%), vomiting status and DKA severity. This model addresses the PH violation and provides valid estimates for the remaining covariates.
2. **Granulated Cox model**: A standard Cox model with DKA severity included as a categorical covariate (mild, moderate, severe). This model estimates the independent effects of all covariates, including DKA severity and serves as the primary analysis.

This approach allows us to both address the PH violation (stratified model) and estimate the independent effects of all covariates (granulated model). The proportional hazards assumption was re‐tested on the stratified model and was satisfied (global test *p* = 0.584).

Hazard ratios (HR) and 95% confidence intervals (CI) are reported. An HR < 1 indicates longer time to discharge (slower discharge) and HR > 1 indicates shorter hospital stay.

All analyses were performed using Stata 18 (StataCorp, College Station, TX). Statistical significance was defined as *p* < 0.05 (two‐tailed). The study followed STROBE guidelines for reporting cohort studies (Table S3). Sensitivity analyses are presented in Table S2.

#### 2.5.5 Ethics Statement

This study received ethical approval from Cavendish University Zambia Research Ethics Committee (Reference: CUZ‐REC/2024/001) and was granted permission to undertake the study by University Teaching Hospital Management (Reference: UTH/ADM/12/2024). A waiver of informed consent was granted for this retrospective analysis of anonymized medical records. All patient identifiers were removed at data extraction, with unique study codes replacing personal information. Data were stored on password‐protected, encrypted devices with access limited to study investigators. The study adhered to principles of the Declaration of Helsinki and complied with the Zambian Data Protection Act, 2021.

## 3 Results

### 3.1 Baseline Characteristics of the Study Population

A total of 353 children with new‐onset diabetic ketoacidosis (DKA) were included in the final analysis. Table 1 summarises the baseline characteristics of the study population stratified by DKA severity (mild vs. moderate/severe).

**TABLE 1:** Baseline characteristics of children with new‐onset DKA (*N* = 353).

Characteristic	Mild DKA (*n* = 101)	Moderate DKA (*n* = 73)	Severe DKA (*n* = 179)	Total (*N* = 353)
N (sample size)	101 (28.6%)	73 (20.7%)	179 (50.7%)	353 (100.0%)
**Age groups**				
≤ 5 years	28 (27.7%)	22 (30.1%)	50 (27.9%)	100 (28.3%)
6–10 years	40 (39.6%)	29 (39.7%)	70 (39.1%)	139 (39.4%)
11–16 years	33 (32.7%)	22 (30.2%)	59 (33.0%)	114 (32.3%)
**Sex**				
Female	50 (49.5%)	36 (49.3%)	87 (48.6%)	173 (49.0%)
Male	51 (50.5%)	37 (50.7%)	92 (51.4%)	180 (51.0%)
**Residence**				
Rural	47 (46.5%)	35 (47.9%)	84 (46.9%)	166 (47.0%)
Urban	54 (53.5%)	38 (52.1%)	95 (53.1%)	187 (53.0%)
**Vomiting**				
No	65 (64.4%)	34 (46.6%)	83 (46.4%)	182 (51.6%)
Yes	36 (35.6%)	39 (53.4%)	96 (53.6%)	171 (48.4%)
**Polyuria**				
No	19 (18.8%)	12 (16.4%)	24 (13.4%)	55 (15.6%)
Yes	82 (81.2%)	61 (83.6%)	155 (86.6%)	298 (84.4%)
**Infection**				
No	66 (65.4%)	44 (60.3%)	105 (58.7%)	215 (60.9%)
Yes	35 (34.6%)	29 (39.7%)	74 (41.3%)	138 (39.1%)
**Family history of T1DM**				
No	77 (76.2%)	57 (78.1%)	140 (78.2%)	274 (77.6%)
Yes	24 (23.8%)	16 (21.9%)	39 (21.8%)	79 (22.4%)
**Ketonuria**				
Negative	40 (39.6%)	12 (16.4%)	38 (21.2%)	90 (25.5%)
Positive	61 (60.4%)	61 (83.6%)	141 (78.8%)	263 (74.5%)
**HbA1c (%), mean (SD)**	10.2 (1.8)	12.7 (2.1)	13.3 (2.2)	11.8 (2.4)
**Hospital stay (days), median (IQR)**	4 (3–5)	6 (4–9)	7 (5–10)	5 (4–8)

Abbreviations: DKA, diabetic ketoacidosis; IQR, interquartile range; SD, standard deviation; T1DM, type 1 diabetes mellitus.

The mean age of participants was 8.3 years (SD 4.6). Slightly more than half were male (180, 51.0%) and just over half resided in urban areas (187, 53.0%). Moderate or severe DKA was present in 179 children (50.7%), while 174 (49.3%) presented with mild DKA.

Vomiting was present in 171 children (48.4%) and was more common in the moderate and severe DKA group (60.9%) compared with the mild DKA group (35.6%). Polyuria was nearly universal, reported in 298 children (84.4%). Infection at presentation was documented in 138 children (39.1%), with a higher proportion in the moderate and severe DKA group (43.6% vs. 34.5%). A family history of type 1 diabetes mellitus was reported in 79 children (22.4%), with similar distribution across severity groups.

Positive ketonuria was present in 263 children (74.5%), and as expected, was more frequent in the moderate and severe DKA group (87.7%) compared with the mild DKA group (60.9%).

The mean HbA1c at presentation was 11.8% (SD 2.4). Children with moderate and severe DKA had higher HbA1c levels (mean 13.1%, SD 2.2) compared with those with mild DKA (mean 10.2%, SD 1.8).

The median hospital stay for the entire cohort was 5 days (IQR 4–8). Children with moderate and severe DKA had a median hospital stay of 7 days (IQR 5–10) compared with those with mild DKA (4 days, IQR 3–5).

### 3.2 Kaplan–Meier Analysis With Log‐Rank Tests

**FIGURE 1:**
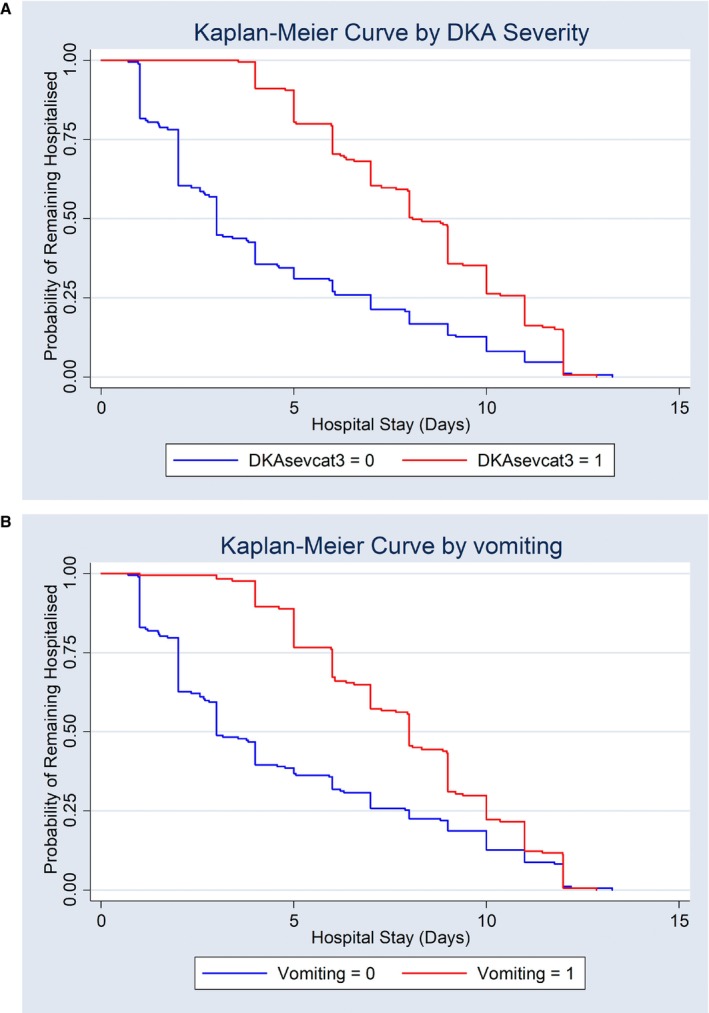
(A) Kaplan–Meier curves for discharge probability by DKA severity. (B) Kaplan–Meier curves for discharge probability by vomiting status.

Figure 1A shows the Kaplan–Meier curves for discharge probability by DKA severity. Children with moderate and severe DKA had consistently lower discharge probability throughout the follow‐up period compared to those with mild DKA. The log‐rank test confirmed a statistically significant difference between the two groups (χ^2^ = 70.32, *p* < 0.001). Children with moderate and severe DKA had a median stay of 7 days compared to 4 days for those with mild DKA.

Figure 1B shows the Kaplan–Meier curves for discharge probability by vomiting status. Children presenting with vomiting had significantly lower discharge probability compared to those without vomiting. The log‐rank test was strongly significant (χ^2^ = 30.35, *p* < 0.001). Children with vomiting had a median stay of 7 days compared to 4 days for those without vomiting.

These unadjusted findings confirm that both DKA severity and vomiting are associated with prolonged hospitalisation before adjustment for other covariates.

### 3.3 Testing the Proportional Hazards Assumption

Before fitting the multivariable model, we formally tested the proportional hazards assumption using Schoenfeld residuals [13].

**Standard Cox model assessment:**

- Global test: χ^2^ = 95.64, df = 10, *p* < 0.001
- Individual covariate tests: HbA1c (*p* = 0.0016), vomiting (*p* < 0.001), DKA severity (*p* = 0.0012) indicating non‐proportionality

To address this violation, we fitted two complementary models:

1. **Stratified Cox model**: Stratified by HbA1c categories (tertiles: ≤ 9.5%, 9.6%–13.0%, > 13.0%), vomiting status and DKA severity.

○ Global test: χ^2^ = 5.62, df = 7, *p* = 0.584
○ Individual covariate tests: all non‐significant (*p* > 0.05)

The stratified Cox model (Table S1) confirmed that the proportional hazards assumption was satisfied (global test *p* = 0.584).

2. **Granulated Cox model**: A standard Cox model with DKA severity as a categorical covariate (mild, moderate, severe) to estimate independent effects.

The stratified model confirmed that the proportional hazards assumption was satisfied (global test *p* = 0.584). The granulated model serves as the primary analysis for estimating independent effects of all covariates.

### 3.4 Granulated Cox Model (Primary Analysis)

The granulated Cox model was statistically significant (likelihood ratio χ^2^ = 356.86, *p* < 0.001). Results are summarised in Table 2.

**TABLE 2:** Granulated Cox regression analysis for time to hospital discharge among children with new‐onset DKA (*N* = 353).

Variable	HR (95% CI)	*p*
**Sex** (ref: Female)		
Male	0.97 (0.78–1.21)	0.80
**Age (years)**	1.00 (0.97–1.02)	0.76
**Family history T1DM** (ref: No)		
Yes	0.89 (0.72–1.11)	0.29
**Polyuria** (ref: No)		
Yes	1.06 (0.85–1.31)	0.63
**Ketonuria** (ref: Negative)		
Positive	0.90 (0.43–1.87)	0.77
**Infection** (ref: No)		
Yes	**1.51 (1.21–1.89)**	**< 0.001**
**Residence** (ref: Rural)		
Urban	0.97 (0.78–1.21)	0.80
**HbA1c categories** (ref: ≤ 9.5%)		
9.6%–13.0%	0.74 (0.54–1.00)	0.05
> 13.0%	**0.66 (0.45–0.96)**	**0.03**
**Vomiting** (ref: No)		
Yes	1.02 (0.78–1.33)	0.91
**DKA severity** (ref: Mild)		
Moderate	**0.01 (0.01–0.02)**	**< 0.001**
Severe	**0.01 (0.01–0.02)**	**< 0.001**

*Note:* HR < 1 indicates slower discharge (prolonged hospital stay). HR > 1 indicates faster discharge. Bold indicates statistical significance at *p* < 0.05.

**Factors associated with delayed discharge (HR** < **1):**

- Moderate DKA (vs mild): HR = 0.01 (95% CI: 0.01–0.02), *p* < 0.001
- Severe DKA (vs mild): HR = 0.01 (95% CI: 0.01–0.02), *p* < 0.001
- HbA1c > 13.0% (vs ≤ 9.5%): HR = 0.66 (95% CI: 0.45–0.96), *p* = 0.028

**Factors associated with faster discharge (HR** > **1):**

- Infection at presentation: HR = 1.51 (95% CI: 1.21–1.89), *p* < 0.001

**Non‐significant factors:**

- Male sex: HR = 0.97 (95% CI: 0.78–1.21), *p* = 0.795
- Age (per year): HR = 1.00 (95% CI: 0.97–1.02), *p* = 0.762
- Family history of T1DM: HR = 0.89 (95% CI: 0.72–1.11), *p* = 0.291
- Polyuria: HR = 1.06 (95% CI: 0.85–1.31), *p* = 0.627
- Ketonuria (positive vs. negative): HR = 0.90 (95% CI: 0.43–1.87), *p* = 0.770
- Urban residence: HR = 0.97 (95% CI: 0.78–1.21), *p* = 0.797
- HbA1c 9.6%–13.0%: HR = 0.74 (95% CI: 0.54–1.00), *p* = 0.051
- Vomiting: HR = 1.02 (95% CI: 0.78–1.33), *p* = 0.906

### 3.5 Stratified Model (PH Assumption Validation)

The stratified Cox model (Table S1) confirmed that the proportional hazards assumption was satisfied after stratification by HbA1c categories, vomiting status and DKA severity (global test *p* = 0.584). In this model, ketonuria appeared significant (HR = 0.21, *p* < 0.001), but this reflects its role as a marker of DKA severity rather than an independent effect, as demonstrated by the granulated model.

A sensitivity analysis using a three‐category DKA severity variable (mild, moderate, severe) confirmed a dose–response relationship (Table S2). The sensitivity analysis using a three‐category DKA severity variable confirmed a dose–response relationship, with moderate (HR = 0.71, 95% CI: 0.53–0.95, *p* = 0.022) and severe (HR = 0.54, 95% CI: 0.40–0.73, *p* < 0.001) DKA showing progressively longer hospital stays compared to mild DKA (Table S2).

## 4 Discussion

This study evaluated determinants of time to hospital discharge among children presenting with new‐onset diabetic ketoacidosis at a tertiary referral hospital in Zambia. Using Kaplan–Meier methods with log‐rank testing and two complementary Cox proportional hazards models a stratified model to address PH violation and a granulated model to estimate independent effects we found that DKA severity is the strongest independent predictor of prolonged hospital stay, while infection at presentation is independently associated with faster discharge [14].

### 4.1 DKA Severity as the Primary Driver of Prolonged Hospital Stay

The granulated Cox model clearly demonstrates that DKA severity is the strongest predictor of delayed discharge. Children with moderate DKA had a 99% reduction in the hazard of discharge compared to those with mild DKA (HR = 0.01, *p* < 0.001), and children with severe DKA had a similarly strong effect (HR = 0.01, *p* < 0.001). This finding aligns with studies from Ethiopia where DKA severity was identified as an independent predictor of recovery time [15], and with other paediatric DKA studies demonstrating that severe metabolic derangement requires prolonged intravenous insulin therapy, aggressive fluid and electrolyte correction and close monitoring for complications such as cerebral oedema [4].

The median hospital stay of 7 days for children with moderate and severe DKA compared with 4 days for those with mild DKA reflects a clinically meaningful difference of 3 days. This has significant implications for resource utilisation, bed occupancy and family financial burden in a low‐resource setting [16].

The sensitivity analysis using a three‐category DKA severity variable confirmed a dose–response relationship, with moderate (HR = 0.71, *p* = 0.022) and severe (HR = 0.54, *p* < 0.001) DKA showing progressively longer hospital stays compared to mild DKA (Table S2).

### 4.2 Ketonuria as a Marker, Not an Independent Predictor

A key insight from our analysis is the distinction between ketonuria and DKA severity. In the stratified Cox model, positive ketonuria appeared to be a strong predictor of delayed discharge (HR = 0.21, 95% CI: 0.10–0.44, *p* < 0.001). However, when DKA severity was included as a covariate in the granulated model, ketonuria lost significance (HR = 0.90, 95% CI: 0.43–1.87, *p* = 0.770). This indicates that **ketonuria is a marker of DKA severity rather than an independent predictor** of prolonged hospital stay.

This distinction is clinically important: it suggests that the presence of ketonuria is not itself the cause of prolonged hospitalisation, but rather reflects the severity of the underlying DKA. Clinicians should focus on DKA severity as the primary driver of prolonged hospital stay, with ketonuria serving as a useful bedside indicator of severity. This is biologically plausible, as ketone clearance requires adequate insulinisation, rehydration and carbohydrate intake all of which take time to achieve [17, 18]. Consistent with the reviewer's observation, we recognise that ketonuria may persist after DKA resolution and is therefore not a sensitive marker of DKA clearance. Our analysis similarly shows that ketonuria is a marker of DKA severity rather than an independent predictor of discharge timing, as it lost significance after adjustment for DKA severity in the granulated model.

### 4.3 Infection and Faster Discharge

Infection at presentation was independently associated with faster discharge (HR = 1.51, 95% CI: 1.21–1.89, *p* < 0.001). This paradoxical finding warrants careful interpretation. Several explanations are possible:

1. **Earlier healthcare‐seeking behaviour:** Children with febrile illnesses may prompt earlier medical attention, leading to shorter duration of untreated diabetes and less severe metabolic decompensation.
2. **Mild infections:** The infections identified in this cohort were predominantly mild (e.g., upper respiratory tract infections) and rapidly responsive to treatment, potentially shortening the overall hospital stay.
3. **Residual confounding:** Despite multivariable adjustment, unmeasured factors such as socioeconomic status or caregiver health literacy could influence both healthcare‐seeking behaviour and discharge timing.

A similar paradoxical finding has been reported in other paediatric DKA studies, where the presence of infection was associated with delayed resolution [19]. However, this finding should not be interpreted as implying that infection is protective. Clinicians should continue to treat infections aggressively and not alter discharge planning based solely on the presence of infection.

### 4.4 Vomiting and HbA1c

While vomiting was a strong predictor in unadjusted analysis (log‐rank χ^2^ = 30.35, *p* < 0.001), it lost significance in the granulated model (HR = 1.02, *p* = 0.906). This suggests that the effect of vomiting on discharge time is largely explained by its association with DKA severity. Vomiting is a common manifestation of severe metabolic acidosis and dehydration in DKA, and its apparent effect is captured by DKA severity itself [20].

Higher HbA1c at presentation (> 13.0% vs. ≤ 9.5%) was independently associated with slower discharge (HR = 0.66, *p* = 0.028), reflecting a longer duration of unrecognised hyperglycaemia and greater baseline metabolic derangement [19]. The borderline significance of the 9.6%–13.0% category (HR = 0.74, *p* = 0.051) suggests a dose–response relationship.

### 4.5 Non‐Significant Factors

Age, sex, polyuria, family history of T1DM and residence were not significantly associated with time to discharge in the granulated model. This suggests that clinical and metabolic factors at presentation are more important determinants of recovery duration than demographic characteristics alone. The borderline significance of family history (*p* = 0.060) in the stratified model suggests a potential protective effect that may become significant in larger studies [16].

### 4.6 Proportional Hazards Assumption

A key strength of our analytical approach is the formal verification of the proportional hazards assumption. The standard Cox model showed significant PH violation (global test *p* < 0.001), which we addressed using stratification. The stratified model showed a non‐significant global test (χ^2^ = 5.62, df = 7, *p* = 0.584), confirming that the PH assumption was satisfied. This methodological rigour increases confidence in the validity of our effect estimates [14].

### 4.7 Comparison With Existing Literature

It is important to clarify that some of the studies referenced in this discussion reported time to DKA resolution (i.e., time to correction of metabolic acidosis) rather than time to hospital discharge [19, 20]. While these are related outcomes, they are distinct measures: time to resolution reflects biochemical recovery, whereas time to discharge incorporates additional clinical and social factors such as readiness for oral intake, caregiver education and availability of follow‐up care. The differences in recovery times between our study and those from Ethiopia may therefore reflect variations in the outcomes measured, in addition to differences in clinical practice and healthcare systems.

Our finding that DKA severity is the strongest predictor of prolonged hospitalisation is consistent with studies from Ethiopia and other settings. A study from Addis Ababa reported that DKA severity was an independent predictor of recovery time (AHR = 2.35, 95% CI: 1.34–6.1) [15]. Similarly, a study from Dessie, Ethiopia found that mild DKA was significantly associated with shorter resolution time [19].

The finding that infection is associated with faster discharge contrasts with some studies. For example, a study from Ethiopia reported that presence of infection was associated with a longer time to resolution of DKA (AHR = 0.65; 95% CI: 0.47–0.90) [19]. This difference may reflect variations in infection severity, healthcare‐seeking behaviour or documentation practices across settings.

The median hospital stay of 5 days (IQR 4–8) in our cohort is shorter than the 8 ± 6.2 days reported in a study from Bahir Dar, Ethiopia [16], but longer than the 27 h median time to resolution reported in Addis Ababa [15]. These differences likely reflect variations in clinical practice, discharge criteria and healthcare systems across settings. A systematic review and meta‐analysis of DKA recovery times in Africa reported a pooled median recovery time of 38 h (95% CI: 33–43 h) [21], highlighting the substantial heterogeneity across the continent. Other Ethiopian studies have reported mean hospital stays of 4.64 days [22] and median resolution times ranging from 24 to 42 h [19, 20], further illustrating the variability in DKA recovery metrics across different healthcare settings.

### 4.8 Strengths

This study is among the first from Sub‐Saharan Africa to apply Cox proportional hazards regression with formal testing of model assumptions to examine time to discharge in paediatric DKA. The use of both stratified and granulated models provides a comprehensive analytical approach the stratified model addresses the PH violation, while the granulated model estimates the independent effects of all covariates. The large sample size and complete follow‐up (no loss to follow‐up) strengthen the internal validity of the findings.

### 4.9 Limitations

Several limitations should be acknowledged. The retrospective design limited control over data completeness and quality. Data on the timing and completeness of diabetes education, family hesitancy regarding insulin administration and separate duration of ICU versus ward stay were not consistently documented, limiting our ability to examine these factors as potential predictors of prolonged hospitalisation. Serum pH and bicarbonate levels were not consistently available, precluding detailed acid–base characterisation. The single centre nature of the study may limit generalisability to district or primary‐level facilities. Unmeasured confounders, including socioeconomic status and caregiver health literacy, could influence both presentation delay and discharge timing. All patients were managed according to a standardised DKA protocol adapted from ISPAD guidelines, which includes intravenous fluid resuscitation, continuous insulin infusion (0.1 units/kg/h) and electrolyte replacement. However, individual variations in practice and precise documentation of insulin infusion rates were not consistently available, precluding analysis of this factor. Additionally, data on anthropometric measures (nutritional status), serum potassium levels, features of raised intracranial pressure, history of prior DKA admissions, time to insulin initiation and persistence of metabolic acidosis were not consistently documented, limiting our ability to evaluate these as potential predictors. Furthermore, infection severity was not consistently graded in the medical records, limiting our ability to examine the relationship between infection severity and length of hospital stay. Data on antibiotic administration or its duration was not collected, which is a limitation. Patients with severe DKA (pH < 7.1) or altered consciousness were admitted to the paediatric ICU, while the majority were managed on the general paediatric ward. However, the duration of ICU versus ward stay was not consistently documented. Complications such as acute kidney injury and cerebral oedema, which could prolong hospital stay, were not consistently documented or evaluated in the records, limiting our ability to examine their impact on discharge timing. The absence of censoring (all patients were discharged) means our findings apply only to survivors and may not capture determinants of more severe outcomes.

### 4.10 Implications for Clinical Practice and Policy

Revisiting the economic rationale that motivated this study, the finding that DKA severity is the principal determinant of prolonged hospital stay has important implications for reducing the financial burden on families and the healthcare system. Strategies to reduce the incidence and severity of DKA at presentation should focus on interventions at the primary and secondary healthcare levels. These include public awareness campaigns to promote early recognition of diabetes symptoms (polyuria, polydipsia and weight loss), training for primary healthcare workers to identify and refer children with suspected diabetes promptly and strengthening referral pathways to tertiary centres. At the community level, social protection measures such as transport vouchers, accommodation support for caregivers and income protection schemes could help mitigate the financial burden of prolonged hospitalisation in economically disadvantaged families. Such measures would not only reduce the direct costs of hospitalisation but also address indirect costs such as lost wages and travel expenses, making paediatric diabetes care more equitable and accessible.

### 4.11 Future Research

The findings of this study generate several important hypotheses and highlight critical knowledge gaps that warrant further investigation.

**Prospective validation:** The predictors identified DKA severity, ketonuria, infection and HbA1c require prospective validation in multicentre studies with standardised documentation of anthropometry, serum potassium, neurological status, insulin timing, metabolic resolution and complications such as AKI and cerebral oedema.

**Interventional trials:** Randomised controlled trials are needed to evaluate whether early nutritional support, structured care pathways, point‐of‐care ketone monitoring or early endocrinology consultation can reduce hospital stays in high‐risk children.

**Health systems research:** Studies exploring caregiver awareness, primary healthcare capacity, referral pathways and cost‐effectiveness of early diagnosis programmes are critical to addressing the underlying systemic barriers to timely DKA diagnosis in Zambia.

**Long‐term outcomes:** Longitudinal studies examining glycaemic control, diabetes complications, psychosocial well‐being and mortality following severe DKA at diagnosis are needed.

**Regional collaboration:** Multicentre networks across sub‐Saharan Africa are essential to pool data, develop context‐specific guidelines, build research capacity and generate regionally relevant evidence to inform policy.

## 5 Conclusion

In this retrospective cohort study of 353 children with new‐onset DKA in Zambia, DKA severity was the strongest independent predictor of prolonged hospital stay. Children with moderate or severe DKA had a median hospital stay of 7 days compared with 4 days for those with mild DKA a difference of 3 days with substantial implications for healthcare resource utilisation and family financial burden in a low‐resource setting. These findings underscore the importance of early diagnosis and prompt management to reduce DKA severity and its consequences. Simple bedside variables, including DKA severity and ketonuria, can guide risk stratification and inform discharge planning in resource‐limited settings. Future prospective studies are needed to evaluate whether targeted interventions at primary and secondary healthcare levels can reduce DKA severity and the associated burden of prolonged hospitalisation.

## Author Contributions

**Martin Kampamba:** writing – review and editing, validation, methodology. **Chanda Chisunka:** methodology, visualization, writing – review and editing, validation. **Gunet Mwalungali:** conceptualization, methodology, software, formal analysis, writing – original draft, validation, writing – review and editing. **Blessings Kaimbo:** conceptualization, data curation, writing – original draft, investigation. **Annie Silwimba:** methodology, investigation, writing – review and editing, validation. **Christine Mutati:** investigation, validation, writing – review and editing. **Larry Mweetwa:** investigation, visualization, validation, writing – review and editing. **Jimmy Hangoma:** validation, writing – review and editing, visualization.

## Funding

No external funding was received for this study. The supporting source had no involvement in study design; collection, analysis or interpretation of data; writing of the report; or the decision to submit the report for publication.

## Disclosure

The lead author and manuscript guarantor, Gunet Mwalungali, affirms that this manuscript is an honest, accurate and transparent account of the study being reported; that no important aspects of the study have been omitted; and that any discrepancies from the study as planned have been explained.

## Ethics Statement

This study received ethical approval from Cavendish University Zambia Research Ethics Committee (Reference: CUZ‐REC/2024/001) and University Teaching Hospital Administration (Reference: UTH/ADM/12/2024) granted us permission to undertake the study. A waiver of informed consent was granted for this retrospective analysis of anonymized medical records. All patient identifiers were removed at data extraction, with unique study codes replacing personal information. Data were stored on password‐protected, encrypted devices with access limited to study investigators. The study adhered to principles of the Declaration of Helsinki and complied with the Zambian Data Protection Act, 2021.

## Consent

The authors have nothing to report.

## Conflicts of Interest

The authors declare no conflicts of interest.

## Supporting information


**Table S1:** Stratified Cox Model (PH Assumption Validation).
**Table S2:** Sensitivity analysis: Three‐category DKA severity model.
**Table S3:** STROBE 2007 (v4) Statement‐Checklist of items that should be included in reports of *cohort studies*.

## Data Availability

The datasets used and analysed during the current study are available from the corresponding author upon reasonable request. Restrictions apply to the availability of these data due to ethical and privacy considerations.
