# Supplementary material for: Clinical Predictors of Time to Hospital Discharge in Children With New‐Onset Diabetic Ketoacidosis in Zambia: A Retrospective Cohort Study
Source: Endocrinol Diabetes Metab. 2026 Aug 12;9(5):e70308. doi: 10.1002/edm2.70308 (PMC13463740; doi:10.1002/edm2.70308)
Supplement: Supplementary file 1 [file EDM2-9-e70308-s001.docx]

**Clinical Predictors of Time to Hospital Discharge in Children with New-Onset Diabetic Ketoacidosis in Zambia: A Retrospective Cohort Study**

**Contents**

1. Supplementary Table S1: Stratified Cox Model (PH Assumption Validation)
2. Supplementary Table S2: Sensitivity Analysis (Three-Category DKA Severity)
3. STROBE Checklist
1. **Supplementary Table S1. Stratified Cox regression analysis for time to hospital discharge among children with new-onset DKA (N = 353)**

| **Variable** | **HR (95% CI)** | **p-value** |
| --- | --- | --- |
| **Sex** (ref: Female) |  |  |
| Male | 0.92 (0.73–1.15) | .449 |
| **Age (years)** | 0.99 (0.97–1.01) | .428 |
| **Family history T1DM** (ref: No) |  |  |
| Yes | 0.81 (0.65–1.01) | .060 |
| **Polyuria** (ref: No) |  |  |
| Yes | 0.92 (0.74–1.15) | .480 |
| **Ketonuria** (ref: Negative) |  |  |
| Positive | **0.21 (0.10–0.44)** | **< .001** |
| **Infection** (ref: No) |  |  |
| Yes | **1.34 (1.07–1.69)** | **.011** |
| **Residence** (ref: Rural) |  |  |
| Urban | 1.05 (0.83–1.32) | .693 |

*Stratified by HbA1c categories (tertiles: ≤9.5%, 9.6–13.0%, >13.0%), vomiting status, and DKA severity.*

**Note:** This model addresses the proportional hazards violation. However, because DKA severity is stratified, its effect is not estimated. The granulated model (Table 2-in the main manuscript) provides the independent effect of DKA severity. The global Schoenfeld test was non-significant (χ² = 5.62, df = 7, p = .584), confirming that the proportional hazards assumption was satisfied.

**Supplementary Table S2. Sensitivity analysis: Three-category DKA severity model**

| **DKA Severity** | **HR (95% CI)** | **p-value** |
| --- | --- | --- |
| Mild (reference) | 1.00 | — |
| Moderate | 0.71 (0.53-0.95) | .022 |
| Severe | 0.54 (0.40-0.73) | < .001 |

*Adjusted for sex, age, HbA1c, family history, polyuria, vomiting, ketonuria, infection, and residence.*

**Note**: To assess the robustness of our findings, we repeated the Cox regression analysis using a three-category DKA severity variable (mild, moderate, severe) instead of the binary classification used in the main analysis. The results demonstrate a clear dose-response relationship: increasing DKA severity is associated with progressively longer hospital stay (moderate: HR = 0.71, p = .022; severe: HR = 0.54, p < .001). This confirms that the main findings are robust to the categorisation of DKA severity.

**Supplementary Table S3. STROBE 2007 (v4) Statement-Checklist of items that should be included in reports of *cohort studies***

| Section/Topic | Item # | Recommendation | Reported on page # | Detailed Response |
| --- | --- | --- | --- | --- |
| **Title and abstract** | 1a | Indicate the study's design with a commonly used term in the title or the abstract | **Page 1** | The title explicitly states "A Retrospective Cohort Study" |
|  | 1b | Provide in the abstract an informative and balanced summary of what was done and what was found | **Page 2** | Abstract summarises background, methods (Kaplan-Meier, Cox regression, stratified/granulated models), key results (HRs, 95% CIs, p-values), and conclusions |
| **Introduction** | 2 | Explain the scientific background and rationale for the investigation being reported | **Page 3** | Background covers global T1DM incidence, DKA burden in LMICs, challenges in Zambia, and rationale for studying time to discharge |
|  | 3 | State specific objectives, including any prespecified hypotheses | **Page 3** | Final paragraph states objective: to identify determinants of time to hospital discharge using KM methods, log-rank tests, and Cox regression with PH testing |
| **Methods** | 4 | Present key elements of study design early in the paper | **Page 4** | "Study Design and Setting" section clearly states retrospective cohort study design |
|  | 5 | Describe the setting, locations, and relevant dates, including periods of recruitment, exposure, follow-up, and data collection | **Page 4** | UTH, Lusaka, Zambia; study period 1 January 2018 to 31 December 2023 |
|  | 6a | Give the eligibility criteria, and the sources and methods of selection of participants | **Page 4-5** | Inclusion criteria (age 0-16, new T1DM diagnosis, documented DKA, complete records) and exclusion criteria (pre-existing diabetes, incomplete records, transfer) clearly stated |
|  | 6b | For matched studies, give matching criteria and number of exposed and unexposed | **Not applicable** | This was not a matched study |
|  | 7 | Clearly define all outcomes, exposures, predictors, potential confounders, and effect modifiers. Give diagnostic criteria, if applicable | **Page 5** | Outcome (time to discharge), exposures (DKA severity, vomiting, infection, ketonuria, HbA1c), predictors, confounders, and ISPAD diagnostic criteria for DKA all defined |
|  | 8 | For each variable of interest, give sources of data and details of methods of assessment (measurement) | **Page 5** | Data extracted from medical records using standardised form; variables categorised (e.g., DKA severity mild/moderate/severe based on ISPAD guidelines) |
|  | 9 | Describe any efforts to address potential sources of bias | **Page 23** | Limitations section acknowledges retrospective design, incomplete data, single-centre nature, unmeasured confounders, and absence of censoring |
|  | 10 | Explain how the study size was arrived at | **Page 4-5** | All eligible cases during study period were included (census approach); no formal sample size calculation |
|  | 11 | Explain how quantitative variables were handled in the analyses. If applicable, describe which groupings were chosen and why | **Page 7** | HbA1c categorised into tertiles (≤9.5%, 9.6–13.0%, >13.0%); DKA severity as mild/moderate/severe based on ISPAD criteria |
|  | 12a | Describe all statistical methods, including those used to control for confounding | **Pages 7** | KM curves with log-rank tests; Cox regression; stratified model (for PH violation); granulated model (for independent effects); PH testing using Schoenfeld residuals |
|  | 12b | Describe any methods used to examine subgroups and interactions | **Not applicable** | No subgroup or interaction analyses performed |
|  | 12c | Explain how missing data were addressed | **Page 4** | Complete-case analysis; patients with incomplete records were excluded (no missing data in final dataset) |
|  | 12d | If applicable, explain how loss to follow-up was addressed | **Page 4** | No loss to follow-up occurred; all patients were discharged (no censoring) |
|  | 12e | Describe any sensitivity analyses | **Page 7** | Sensitivity analysis using three-category DKA severity (mild, moderate, severe) to assess robustness of findings; results in Supplementary Table S2 |
| **Results** | 13a | Report numbers of individuals at each stage of study-e.g., numbers potentially eligible, examined for eligibility, confirmed eligible, included in the study, completing follow-up, and analysed | **Page 8** | 353 children included in final analysis; all completed follow-up (discharged) |
|  | 13b | Give reasons for non-participation at each stage | **Page 4-5** | Exclusion criteria: pre-existing diabetes, incomplete records, transfer to another facility before discharge |
|  | 13c | Consider use of a flow diagram | **Not applicable** | Flow diagram not included; all cases easily described in text |
|  | 14a | Give characteristics of study participants (e.g., demographic, clinical, social) and information on exposures and potential confounders | **Pages 7-8** | Table 1 summarises age, sex, residence, vomiting, polyuria, infection, family history, ketonuria, HbA1c, and hospital stay stratified by DKA severity |
|  | 14b | Indicate number of participants with missing data for each variable of interest | **Page 4** | Complete-case analysis; no missing data in final dataset (patients with incomplete records excluded) |
|  | 14c | Summarise follow-up time (e.g., average and total amount) | **Page 9** | Median hospital stay 5 days (IQR 4–8); Table 1 shows stay by severity: mild 4 days (IQR 3–5), moderate/severe 7 days (IQR 5–10) |
|  | 15 | Report numbers of outcome events or summary measures over time | **Page 8-9** | 353 (100%) patients discharged; median stay reported; KM curves show discharge probability over time |
|  | 16a | Give unadjusted estimates and, if applicable, confounder-adjusted estimates and their precision (e.g., 95% confidence interval). Make clear which confounders were adjusted for and why they were included | **Pages 16** | Table 2 presents unadjusted and adjusted HRs with 95% CIs and p-values; adjusted for sex, age, HbA1c, family history, polyuria, vomiting, DKA severity, ketonuria, infection, residence |
|  | 16b | Report category boundaries when continuous variables were categorized | **Page 7** | HbA1c tertiles: ≤9.5%, 9.6–13.0%, >13.0%; DKA severity: mild, moderate, severe based on ISPAD criteria |
|  | 16c | If relevant, consider translating estimates of relative risk into absolute risk for a meaningful time period | **Not applicable** | HRs are reported; absolute risk not calculated |
|  | 17 | Report other analyses done-e.g., analyses of subgroups and interactions, and sensitivity analyses | **Pages 15, 16,17,18** | Stratified Cox model (Supplementary Table S1) for PH validation; sensitivity analysis using three-category DKA severity (Supplementary Table S2) |
| **Discussion** | 18 | Summarise key results with reference to study objectives | **Page 8-17** | Discussion first paragraph summarises key finding: DKA severity is strongest predictor of prolonged hospital stay; infection associated with faster discharge |
|  | 19 | Discuss limitations of the study, taking into account sources of potential bias or imprecision. Discuss both direction and magnitude of any potential bias | **Page 18** | Limitations: retrospective design, incomplete laboratory data (pH/bicarbonate), single-centre, unmeasured confounders (socioeconomic status, caregiver health literacy), absence of censoring |
|  | 20 | Give a cautious overall interpretation of results considering objectives, limitations, multiplicity of analyses, results from similar studies, and other relevant evidence | **Pages 8-17** | Cautious interpretation throughout; ketonuria identified as marker of DKA severity not independent predictor; infection finding interpreted cautiously |
|  | 21 | Discuss the generalisability (external validity) of the study results | **Page 23** | Single-centre nature may limit generalisability to district or primary-level facilities; findings applicable to tertiary referral settings in similar LMICs |
| **Other information** | 22 | Give the source of funding and the role of the funders for the present study and, if applicable, for the original study on which the present article is based | **Page 26** | No external funding received; supporting source had no involvement in study design, data collection, analysis, writing, or decision to submit |
